# Targeting pleiotropic signaling pathways to control adult cardiac stem cell fate and function

**DOI:** 10.3389/fphys.2014.00219

**Published:** 2014-07-01

**Authors:** Stefania Pagliari, Jakub Jelinek, Gabriele Grassi, Giancarlo Forte

**Affiliations:** ^1^Integrated Center for Cell Therapy and Regenerative Medicine (ICCT), International Clinical Research Center, St. Anne's University HospitalBrno, Czech Republic; ^2^Department of Life Sciences, University of TriesteTrieste, Italy

**Keywords:** cardiac stem cell, heart regeneration, cell signaling, differentiation, stem cell homeostasis

## Abstract

The identification of different pools of cardiac progenitor cells resident in the adult mammalian heart opened a new era in heart regeneration as a means to restore the loss of functional cardiac tissue and overcome the limited availability of donor organs. Indeed, resident stem cells are believed to participate to tissue homeostasis and renewal in healthy and damaged myocardium although their actual contribution to these processes remain unclear. The poor outcome in terms of cardiac regeneration following tissue damage point out at the need for a deeper understanding of the molecular mechanisms controlling CPC behavior and fate determination before new therapeutic strategies can be developed. The regulation of cardiac resident stem cell fate and function is likely to result from the interplay between pleiotropic signaling pathways as well as tissue- and cell-specific regulators. Such a modular interaction—which has already been described in the nucleus of a number of different cells where transcriptional complexes form to activate specific gene programs—would account for the unique responses of cardiac progenitors to general and tissue-specific stimuli. The study of the molecular determinants involved in cardiac stem/progenitor cell regulatory mechanisms may shed light on the processes of cardiac homeostasis in health and disease and thus provide clues on the actual feasibility of cardiac cell therapy through tissue-specific progenitors.

## Introduction

Early pre-clinical and clinical trials involving skeletal myoblasts (Menasché et al., 2008), mesenchymal (Dixon et al., 2009) and hematopoietic stem cells (Balsam et al., 2004; Murry et al., 2004) proved mild, non-reproducible effects on cardiac diseases. In fact, the intracoronary delivery of bone marrow mesenchymal stem cells (MSCs) in patients affected by acute myocardial infarction (AMI) only proved their worth improving left ventricular ejection fraction by 3%, while failing to modify the negative impact of ventricular remodeling. In the case of chronic heart failure (CHF), skeletal myoblast or bone marrow mononuclear cell implantation failed to provide any benefit to the patient, other than a small improvement ascribed to the secretion of beneficial factors. A different scenario appears when the outcome of the pathology can be improved by neo-angiogenesis, like in the case of refractory angina (RA). In this case, bone marrow cells (namely CD34+ hematopoietic progenitors) can provide some degree of endothelial differentiation together with the positive paracrine effect to help the cause (Menasché, 2011).

Anyway, the rate of transdifferentiation obtained by bone marrow or skeletal muscle-derived progenitors is limited. Thus, cardiac resident progenitor cells (CPCs) are considered the elective source of autologous contractile cells for transplantation (Chugh et al., 2012). Among adult cardiac resident stem cells, the few subsets identified on the basis of the expression of c-kit (CD117, Quaini et al., 2002) or Stem Cell Antigen 1 (Sca-1, Bailey et al., 2012) are still questioned for their actual role in contractile tissue homeostasis and regeneration.

Despite the incredible amount of information regarding the role of specific signaling pathways acting during cardiogenesis to finely control cell specification within the forming organ, little is known about the molecular mechanisms governing adult tissue-specific progenitor cell function.

The issue of tissue-resident stem cell fate and function control has been so far addressed by using the instruments and knowledge coming from the study of somatic cells.

Nonetheless, compelling evidence supports the notion that tissue-resident stem cell behavior and fate may be, at least in part, modulated through non-canonical stimuli able to activate cell-specific signaling pathways and in turn leading to a given biological response (Guilak et al., 2009; Kaivosja et al., 2012).

The organogenesis and healing programs present a high degree of complexity because of the precise spatial and temporal activation of signaling pathways that are deemed to interact with each other.

In the present review, we would like to draw the attention on the possibility that the regulation of cardiac resident stem cell fate and function is the result of the interplay between pleiotropic signaling pathways and cell-specific regulators. Thus we summarize the main pathways acknowledged to participate in the regulation of adult CPC fate and function so far.

## Wnt signaling pathway

Many biological processes occurring in cardiac stem cells, including cardiac specification, survival and differentiation, have been associated to Wnt signaling pathway (Flaherty et al., 2012). Wnt signals consist of secreted glycoproteins acting in a paracrine fashion by binding to Frizzled (FZD) family of trans-membrane receptors and activating the so-called canonical or non-canonical transduction pathways.

Different co-receptors are independently activated in response to canonical (LRP-5/LRP-6) or non-canonical (ROR2 and RYK) signaling (Katoh and Katoh, 2007) and intracellular Wnt signaling regulators are also involved in the transduction of one or the other pathway. β-catenin is the main effector of canonical Wnt signaling whereas non-canonical Wnt signals are transduced through the Rho/Rac/JNK-dependent or the calcium-dependent signaling cascades.

Wnt pathways play a role in the development and differentiation of both the embryo and adult heart and are highly interconnected. The nuclear translocation of β-catenin was proven to up-regulate the transcription of genes controlling CPC amplification (e.g., c-myc and cyclin D1). On the other hand, the inhibition of β-catenin-dependent transcription via proteasomal degradation of its cytoplasmic form triggers CPC differentiation toward the contractile phenotype (Bergmann, 2010).

Although no evidence has been provided regarding the activation of β-catenin signaling in the healthy adult heart (Cohen et al., 2008), this pathway is being activated after myocardial infarction (Bergmann, 2010). Interestingly, β-catenin depletion is beneficial to infarcted post-natal mouse heart leading to both attenuated mortality and LV remodeling. It also up-regulates the expression of Tbx5 and GATA4 cardiac lineage markers while enhancing Sca-1+ resident cardiac progenitor cell terminal differentiation (Zelarayan et al., 2008).

Consistently Krueppel-like factor 15 (KLF15)—a transcription factor regulating cardiac β-catenin transcriptional activity—was found to be critical in controlling CPC homeostasis. In fact, the *in vivo* proliferative and cardiogenic potential of Sca-1+ CPCs was reduced in hearts derived from Klf15-/- mice in normal and hypertrophic conditions. Additionally, the activation of β-catenin-dependent transcription upon loss of KLF15 prompted the CPCs toward an endothelial phenotype (Noack et al., 2012).

Following this experimental evidence, Wnt/β-catenin signaling inhibition via the local release of effective Wnt scavengers or inhibitors (e.g., soluble frizzle-related proteins sFRPs and the secreted protein Dkk1) has become an attractive therapeutic target to prevent heart failure and preserve cardiac function.

Moreover the reactivation of Wnt pathway could be explored as a suitable strategy to protect adult c-kit^+^ CPCs from oxidative stress-mediated apoptosis in infarcted heart (Liu et al., 2013) and restore LV function (Bergmann, 2010).

More recently, a biphasic function for Wnt/β-catenin signaling during cardiac development has been also proposed, implying that the effects of Wnt signals (including Wnt1, Wnt2a, Wnt3a, and Wnt8) or their inhibitors can depend on the stage of development (Naito et al., 2006; Tzahor, 2007; Ueno et al., 2007).

Studies on the role of Wnt/β-catenin signaling during cardiac tissue specification demonstrated that the upregulation of Wnt/β-catenin signaling is essential to the formation of mesodermal tissue (Huelsken et al., 2000) and to enhance the cardiac commitment or the expansion of cardiac progenitors during the early phases of cardiogenesis (Qyang et al., 2007; Lindsley et al., 2008).

At a later stage, the inhibition of Wnt/β-catenin signals, mediated by the early cardiac-specific marker Mesp1, is pivotal for the terminal differentiation of precursors into cardiomyocytes (David et al., 2008).

Noteworthy, the Wnt/β-catenin signaling seems also to inhibit early cardiac commitment of progenitors via a non-cell-autonomous mechanism, very likely to be exerted through the release of signals by the associated endoderm in mouse ES cells and embryos (Lickert et al., 2002; Liu et al., 2007). As such, Wnt signals would participate in cardiac specification and differentiation through several phases of activation and inhibition (Gessert and Kuhl, 2010) as well as in the regulation of cell-cell interaction through the involvement of secondary factors acting on cardiac mesoderm formation during heart development. In this respect, the effects of this universal pathway would be the result of its interaction with cell-specific adaptors, thus leading to a context- and cell-specific response.

For instance, a positive effect on the proliferation of neonatal and embryonic Isl-1^+^ CPCs from both *in vivo* and *ex vivo* explants was exerted by Wnt/β-catenin signaling through the downstream activation of multiple FGF ligands, in particular FGF10 (Cohen et al., 2007). In contrast, an excess of β-catenin signaling as a result of the up-regulation of insulin-like growth factor-binding protein 3 (IGFBP3) was associated to the reduction of the proliferative capacity in adult cultured Sca-1+/Islet-1- cardiac stem cells (Oikonomopoulos et al., 2011).

Recently, an increased β-catenin activity mediated by stromal cell derived factor 1α (SDF1α) has been associated with a reduction in c-kit+ CPCs proliferation. This event might restrain heart endogenous reparative response following conditions (stress or injury) characterized by high levels of the cytokine (Dimova et al., 2014). The reduction of proliferation in response to β-catenin activation might be suggestive of cardiac commitment/differentiation of tissue-specific progenitors. In accordance with this hypothesis, loss-of function experiments established an undisclosed relationship between Sca-1 antigen and canonical Wnt signaling in regulating the balance between c-kit+ CPC proliferation and differentiation. In fact, c-kit+ CPCs from Sca-1-knockout models exhibited poor proliferation and increased cardiogenic commitment, very likely due to the activation of β-catenin signaling (Bailey et al., 2012).

In contrast to canonical Wnt signaling, non-canonical pathway is less well characterized and its role in cardiac stem cell biology remains to be elucidated. Several studies have evidenced a positive effect of non-canonical Wnt signaling in mouse ES cell differentiation (Cohen et al., 2008), with Wnt5a and Wnt11 being requested to promote cardiac differentiation through non-canonical pathway in both the embryo and progenitor cells (Eisenberg and Eisenberg, 1999; Pandur et al., 2002; Brade et al., 2006; Palpant et al., 2007; Flaherty and Dawn, 2008; Cohen et al., 2012).

Current evidence suggests that some degree of overlapping exists between the two pathways, although the actual relevance of this interaction in cardiac tissue formation and repair remains poorly understood.

## Notch signaling pathway

Notch pathway is involved in the regulation of tissue homeostasis and formation during embryonic and adult life by controlling cell fate decision and the maintenance and differentiation of stem cells (Nemir and Pedrazzini, 2008). Notch activity participates in cardiogenesis at multiple stages, from the commitment of mesoderm to the maturation of myocytes, in a context- and time-dependent fashion (Gude and Sussman, 2012).

In the developing heart, Notch regulates ventricular trabeculation, cardiomyocyte proliferation and differentiation, as well as valve formation (High and Epstein, 2008).

Interestingly, both the over-expression and inhibition of Notch signaling have been associated with the onset of cardiac defects (Kratsios et al., 2010), pointing at its fine regulation as a key determinant in fetal heart development.

In mammals, Notch pathway mediates the interaction between adjacent cells expressing Notch receptors (Notch1–4) and their ligands (Delta-like 1, 3, 4, and Jagged 1, 2) (Chiba, 2006; Bolós et al., 2007).

Consequently, the fate and function of any given cell are strictly regulated by adhesive intercellular interactions and dependent on the fate of the neighboring cells. Upon the binding of the ligand, Notch intra-cytoplasmic domain (NICD) is released by proteolytic cleavage, can translocate to the nucleus, interact with DNA-binding proteins and participate in the control of downstream effectors.

The most well-known downstream effectors of Notch pathway are Hes and Hes-related (Hesr) family of proteins, acting as repressors of the transcription of Notch-dependent genes (Iso et al., 2003).

In the post-natal heart, Notch1 is the predominant isoform, being expressed in immature cardiomyocytes and non-myocyte cells (Croquelois et al., 2008; Gude et al., 2008; Nemir and Pedrazzini, 2008). The activation of this pathway in adult heart evokes pro-survival, proliferative and anti-apoptotic effects in cardiomyocytes, thus contributing to attenuate cardiac hypertrophy, fibrosis and ameliorate cardiac performance (Croquelois et al., 2008; Nemir et al., 2012).

Together with its target gene Hes-1, Notch pathway was found up-regulated in cardiomyocytes at the border zone of the infarcted region (Gude et al., 2008) and also cardiomyocyte treatment with hepatocyte growth factor (HGF), exerting cardioprotective effects through its receptor c-Met, was found to act through the activation of Notch signaling.

NICD over-expression in the infarcted heart caused functional and morphological improvement, although no information on the possible contribution of resident stem cells to this process was given. Nonetheless, since c-kit+ and Sca-1+ cardiac progenitors express c-Met receptor and secrete HGF (Urbanek et al., 2005), the homeostasis of the adult myocardium might, at least in part, be guided by the activation of Notch pathway in undifferentiated cells homing to the damaged site.

Consistently, evidence is being accumulated suggesting that resident CPC recruitment and differentiation is an effect of Notch-mediated induction of reparative mechanisms in the damaged adult myocardium (Campa et al., 2008; Gude et al., 2008; Kratsios et al., 2010; Li et al., 2010).

Additionally, cycling CPCs displayed high Notch activity as a means to restrict their cardiomyocyte differentiation potential, thus predicting for Notch a role in maintaining the self-renewal and stemness state of the cardiac precursor pool residing in mouse and rat normal adult hearts (Collesi et al., 2008; Croquelois et al., 2008).

This result paralleled those obtained in embryonic stem cells in which the activation of Notch signaling hindered the differentiation of mesodermal progenitors into cardiomyocytes (Nemir et al., 2006; Schroeder et al., 2006). Furthermore, in a late stage of the hypertrophic response, Jagged-1 ligand expression was found up-regulated on the membrane of cardiomyocytes: this event was shown to affect the expansion of the neighboring Notch-positive cardiomyocytes and progenitor cells.

However, these results were obtained in a non-purified population of cardiac stem/progenitor cells, exclusively selected for their lack of markers of terminal differentiation.

The *in vivo* analyses of adult murine cardiac tissue highlighted an interesting pattern in Notch distribution: a baseline expression of Notch-1 receptor in c-kit+ CPCs residing in cardiac niche was confirmed. As expected, these cells are surrounded by cells expressing Jagged-1 ligand. A similar correlation between Notch-1 and Sca-1 expression in stem cell niche has been also suggested (Boni et al., 2008).

Although these data seem to imply a linear dependence of stem cell niche regulation by the surrounding tissue, a more sophisticated Notch-based mechanism has been shown to regulate the balance between the proliferation and differentiation in cardiac stem cells, thus questioning the actual role of Notch on the maintenance of stemness phenotype. In fact, upon Jagged-1-mediated stimulation, Notch-1 activation paralleled the up-regulation of Nkx-2.5, an early regulator of cardiac commitment, driving the cell toward a phenotype of highly proliferative myocytes.

These results would provide evidence of the existence of a modular interplay between the ubiquitous Notch pathway and cell-specific actuators (Nkx-2.5) to evoke a unique response of cardiac progenitors to external stimuli.

In the border zone of the infarcted myocardium, the pharmacological inhibition of Notch reduced the number of cycling c-kit+ CPCs and compromised their cardiac commitment. Accordingly, a severe dilated myopathy developed in newborn mice following the inhibition of Notch signaling, as a consequence of the reduced differentiation rate of cardiac progenitors (Urbanek et al., 2010).

The complexity of Notch impact on cardiac progenitor regulation is demonstrated by its role in epicardial cells. Epicardium has been recently suggested to act as a cardiac stem cell niche contributing to the pool of cardiac progenitors in developing and adult heart (Zhou et al., 2008; Popescu et al., 2009; Di Meglio et al., 2010). Indeed MI causes the activation of epicardium-derived progenitors (Limana et al., 2010) to promote cardiac repair by contributing to neovascularization and generating cardiomyocytes structurally and functionally integrated with the resident myocardium (Smart et al., 2012).

Notch blockade lead to an up-regulation of pluripotency genes in epicardial-derived c-kit+ cells from rat hearts and delayed their differentiation (Zakharova et al., 2012). In particular, Notch signaling was found to favor epithelial-to-mesenchymal transition (EMT) in epicardial precursors in culture.

The transition between an epithelial and mesenchymal cell phenotype is a crucial and reversible process (mesenchymal-to-epithelial transition, MET) operating during embryogenesis and organ development but can also promote the acquisition of stemness properties *in vitro* (Di Meglio et al., 2010; Forte et al., 2012). EMT has been found reactivated in adult cells in pathological conditions (Zeisberg et al., 2007; Thiery et al., 2009) and demonstrated to be crucial in embryonic stem cell differentiation (Martínez-Estrada et al., 2010).

This process is fundamental in the epicardium contributing to coronary vasculature and myocardium formation during heart development (Zhou et al., 2011). At this stage epicardium-derived cells (EPDCs) undergo three successive rounds of EMT and MET to acquire the final differentiated phenotype and spatial organization (Thiery et al., 2009).

Of note, both c-kit+ and c-kit- cardiac progenitor cells migrating out of atrial explants *in vitro* were shown to transiently express epicardial markers and to proceed to EMT in culture. Different signaling pathways belonging to TGFβ superfamily, Wnt/β-catenin, Notch, HGF, FGF, retinoic acid families have been described (von Gise and Pu, 2012; Zakharova et al., 2012) as key players in the fine regulation of EMT process.

## JAK/STAT signaling pathway

The hypothesis that JAK/STAT pathway could be involved in cardiogenesis has been initially explored in Drosophila. Drosophila embryos with targeted mutations in the JAK/STAT ligand or in STAT92E transducer developed non-functional hearts with defects in lumen formation very likely as an effect of inappropriate cell-cell interaction and abnormal cell aggregation. This effect has been associated to JAK/STAT pathway regulation of Tinman protein expression prior to heart precursor cell diversification (Johnson et al., 2011). Tinman is the Drosophila homolog of Nkx-2.5 mammalian transcription factor. Thus, the existence of an interaction between JAK/STAT ubiquitous signaling pathway and cardiac-specific regulators during tissue-specific stem cell specification in mammalians can be inferred, although no evidence has been given so far.

JAK-STAT signaling has been demonstrated to be essential for keeping ES self-renewal and pluripotency (Niwa et al., 1998; Matsuda et al., 1999). The inhibition of JAK2/STAT3 signaling in embryonic bodies prevented the formation of beating areas, while its ectopic expression had opposite effects (Foshay et al., 2005).

The same pathway has been found to protect murine cardiac tissue by different means, the most remarkable being the mobilization of endothelial progenitors from the bone marrow (Mohri et al., 2012). Additionally, a direct effect of this signaling pathway on cardiac cells was proposed. For example, the attenuation of cardiac fibrosis after MI through the activation of STAT3 by IL-11 in cardiac myocytes has been demonstrated (Obana et al., 2010). This cytokine, together with CT-1 was found highly expressed in the heart during myocardial infarction.

STAT3 pathway was also activated in Sca-1+ cardiac progenitor cells treated with IL-11 and CT-1. The result of such stimulation on the cells was the up-regulation of endothelial markers (Mohri et al., 2009). This process is thought to be a pro-angiogenic response of resident stem cells to the hypoxic and inflammatory environment of the post-ischemic area.

Surprisingly, a similar effect could not be evoked by the inflammatory cytokine IL-6 in Sca-1+ cells, very likely because of the lack of gp130 receptor on the membrane of this stem cell subset (Mohri et al., 2012).

On the contrary, JAK-STAT pathway can be activated through IL-6 receptor in c-kit cardiac stem cells, thereby inducing their endothelial differentiation. These effects are likely to reflect the tight association between the inflammatory burst occurring after myocardial infarction and the process of neo-vascularization.

## Hippo pathway in cardiac stem/progenitor cells

The Hippo pathway has been recently identified as a fundamental axis in the regulation of organ size and shape during organogenesis and its involvement in the pathogenesis of cancer has been established (Johnson and Halder, 2014).

This pathway negatively controls the nuclear shuttling of the paralog Yes-associated protein (YAP) and WW domain-containing transcription regulator protein 1 (WWTR1 or TAZ) by sequestering them into the cytoplasm and inducing their degradation through proteasome activity (Hong and Guan, 2012).

The downstream effectors of Hippo pathway have also been identified as mammalian proto-oncogenes.

YAP and TAZ have no transcriptional activity *per se* but function as gene co-activators by binding cell-specific transcription factors (Tian et al., 2010).

YAP/TAZ axis has been described as a central regulator of human embryonic stem cell self-renewal through the control of SMAD complex shuttling to the nucleus, with TAZ knock-down resulting in the loss of cell pluripotency (Varelas et al., 2010). The same co-factors control intestinal (Camargo et al., 2007) and neural progenitor cell number and differentiation (Cao et al., 2008) by targeting the Notch signaling. YAP activity has also been proven fundamental in controlling epidermal stem cell homeostasis (Zhang et al., 2011).

The activity of TAZ as a modulator of MSC differentiation has been shown to direct cell differentiation into osteoblasts and adipocytes through its ability to bind lineage-specific master transcription factors: in fact, MSC fate could be controlled by TAZ ability either to activate Runx2 or to inhibit PPARγ, respectively (Hong et al., 2005; Hong and Yaffe, 2006).

Moreover, the same factor has been demonstrated to be crucial in directing myogenic differentiation by physically associating with MyoD in skeletal muscle progenitors (Jeong et al., 2010).

Yap1 is an important regulator of cardiomyocyte proliferation and embryonic heart development, its lack being associated with diminished cardiomyocyte proliferation (von Gise et al., 2012) and embryonic lethality (Heallen et al., 2011) during cardiogenesis.

In the adult heart, Hippo pathway has been connected to cardiomyocyte response and protection against ischemic injury (Del Re et al., 2013).

Our group recently identified the role of YAP/TAZ proteins as regulators of cardiac stem/progenitor cell sensitivity to the stiffness and nanotopography of the microenvironment (Mosqueira et al., 2014).

By using human and murine cardiac resident stem cells isolated on the basis of Stem Cell Antigen 1 (Sca-1) expression, we demonstrated the ability of YAP and TAZ to translocate from the cytoplasm to the nucleus and back in response to dynamic modifications in substrate elasticity and nanostructure.

Similarly to what was shown in mesenchymal stem cells (Dupont et al., 2011), in Sca-1+ cardiac stem cells, YAP/TAZ re-localization in the nucleus seems to be controlled by the availability of adhesion sites within the extracellular matrix (ECM) and the ability of the cells to spread and acquire a given shape.

Along with their key role in mastering CPC migration, YAP/TAZ participate in fate decision in tripotent Sca-1+ cardiac stem/progenitor cells: its inhibition by gene silencing results in a switch between cardiac and endothelial lineage commitment. According to our unpublished results, a direct interaction could occur in the cytoplasm between YAP/TAZ protein and GATA-4, Tbx-5 cardiac-specific transcription factors. This binding would induce the complex to migrate to the nucleus of Sca-1+ CPCs and trigger specific responses (our unpublished results).

## PI3K-Akt/Pim-1 kinase axis

Akt is a kinase promoting a number of biological functions including cell survival, proliferation, and differentiation (Sussman, 2007). Akt is involved in a number of cell processes as a result of its interaction with different pathways.

The cardiac-specific overexpression of nuclear Akt was found to increase cell cycling in resident cardiac progenitor cells, an effect which has been associated with cardioprotective significance (Fujio et al., 2000; Shiraishi et al., 2004; Gude et al., 2006; Tsujita et al., 2006).

Through ectopic expression experiments, Akt has been deemed responsible of the paracrine effect implanted mesenchymal stem cells exert on injured cardiac tissue. The molecular basis of this effect has been ascribed to the ability of Akt-transduced MSCs to secrete a massive amount of beneficial molecules, namely VEGF, bFGF, HGF, IGF-1, and thymosin β 4 (TB4), thus evoking cardiac tissue remodeling and local neo-vascularization (Gnecchi et al., 2008). In this context, a possible effect of the factors secreted on the resident pool of cardiac stem/progenitor cells has been hypothesized.

Recently, the cooperation between Akt and Pim-1 kinases has been identified in c-kit+ cardiac progenitor cells. Pim-1 is known to exert anti-apoptotic effects on the myocardium as a result of the phosphorylation of Bad and inhibition of caspase cleavage (Muraski et al., 2007).

Also, Pim-1 kinase phosphorylates heterochromatin protein-1 and nuclear mitotic apparatus protein (NuMA), which are crucial in cell division, while promoting cell proliferation in cancer and hematopoietic stem cells (Sun and Schatten, 2006) through the collaboration with c-Myc (Stewart et al., 1999; Zhang et al., 2007, 2008).

In c-kit+ cardiac stem cells, Pim-1 over-expression was found to enhance CPC proliferation through asymmetric division, with Pim-1 transgenic mice displaying a higher proliferative activity in the heart as compared to the controls (Cottage et al., 2010).

A hypothesis on the role of Pim-1 kinase has been formulated in which its overexpression is responsible for CPC increased proliferation during the hyperplastic phases occurring in physiological (during pre- and post-natal development) and in pathological (after myocardial infarction) conditions.

## HGF and IGF1 in cardiac stem cells

The possible role of Hepatocyte Growth Factor (HGF) and Insulin-like Growth Factor-1 (IGF-1) in the homeostasis and differentiation of resident cardiac stem cells has been the subject of a few investigations in which they were studied together.

Hepatocyte growth factor (HGF) is a pleiotropic cytokine of mesenchymal origin, promoting motility, proliferation, invasion, morphogenesis, and survival in a wide spectrum of cells, namely epithelial and endothelial cells (Trusolino and Comoglio, 2002; Birchmeier et al., 2003). HGF controls the tubulogenesis during kidney (Santos et al., 1994) and mammary gland (Yang et al., 1995) development and angiogenesis (Bussolino et al., 1992). The protein, originally identified as scatter factor (SF), acts as a chemoattractant for motor neuron axon (Ebens et al., 1996) and myogenic precursors (Bladt et al., 1995; Maina et al., 1996) and a survival factor for hepatocytes (Schmidt et al., 1995) and placenta (Uehara et al., 1995). Our group identified the presence of HGF putative receptor c-Met on murine mesenchymal stem cells and successfully achieved MSC commitment toward cardiac phenotype through HGF stimulation. The cells failed to acquire a mature cardiomyocyte phenotype by our protocol (Forte et al., 2006). In this study, HGF activity in MSCs was shown to activate the ras-ERK1/2 and p38 MAPKs as well as the PI3K/Akt pathways.

Consistently, HGF has been shown to improve embryonic stem cell differentiation toward the cardiac phenotype (Roggia et al., 2007).

HGF has a cardioprotective role in experimentally induced myocardial infarction, preventing cardiomyocyte apoptosis, inducing angiogenesis, and improving impaired heart function (Aoki et al., 2000; Jayasankar et al., 2003; Sala and Crepaldi, 2011).

c-kit+ cardiac resident stem cells were shown to secrete HGF while expressing its functional receptor on their membrane. The factor acts as a chemoattractant and increases c-kit+ cardiac stem cell invasive ability. This cell subset was shown to express a functional receptor for IGF-1, while its administration had few, if any, motogenic or invasive activity on them. Interestingly, IGF-1 displayed a marked anti-apoptotic activity in CSCs (Urbanek et al., 2005).

Insulin-like growth factor-1 (IGF-1) is a potent mitogen, exerts anti-apoptotic activity, and is necessary for neural stem cell growth (Arsenijevic et al., 2001). IGF-1 also promotes myocyte formation while protecting them from the injury due to myocardial infarction (Li et al., 1997). Moreover, the proliferation and cardiac induction of developing murine mesoderm could be promoted *in vitro* by administering IGF, thus disclosing a novel role of this cytokine in cardiac mesoderm specification (Engels et al., 2014).

Both IGF-1 and HGF, whose expression was found significantly increased in the infarction border zone after myocardial ischemic insult (Fiaccavento et al., 2005), were shown to enhance cardiac stem cell proliferation. The local delivery of both the factors in the infarcted heart resulted in the migration of c-kit+ cardiac stem cells toward the injured area and the regeneration of dead portions of the myocardium (Urbanek et al., 2005).

Moreover, the regenerative response of endogenous porcine c-kit+/CD45− cardiac stem cells to an ischemic insult could be enhanced *in situ* by administering the two growth factors directly through the coronary circulation (Ellison et al., 2011). This treatment resulted in a persistent dose-dependent and positive effect on cardiac performance, probably due both to a direct effect on the survival of the cardiomyocytes and to an enhancement in cardiac stem cell function.

## Examples of the interaction between different pathways in cardiac progenitor cells

The interplay among the molecular pathways taking part to cardiogenesis could be based on the sharing of molecular effectors, with WNT/β-catenin, Hippo, TGF β and BMP signaling being considered pivotal in the specification and maturation of cardiac precursors (Attisano and Wrana, 2013).

An example of such an interplay comes from the use of conditional mutagenesis in mouse embryos. By this means, a hierarchical regulation of cardiac progenitor differentiation has been unveiled, in which WNT/β-catenin acts as downstream effector of Notch signaling and upstream of BMP signaling during embryonic heart development (day 9.0). In this context, progenitor cardiac differentiation requires the sequential expression of distinct sets of cardiac transcription factors controlled by WNT/β-catenin and Bmp4 signaling. At an early stage WNT/β-catenin activates *Baf60c*, *Nkx2-5*, and *Isl1* genes but also BMP4 signaling which, in turn, activates Gata4 and SRF (Klaus et al., 2012).

Similarly, the Hippo pathway effector TAZ has been shown to negatively regulate WNT pathway by inhibiting Disheveled, an upstream inhibitor of the β-catenin destruction complex, thus reducing β-catenin stabilization in human embryonic kidney cells (Varelas et al., 2010). Therefore, Hippo pathway appeared to restrict WNT/β-catenin signaling by TAZ cytoplasmic activity (Varelas et al., 2010). The interaction between Hippo and WNT signaling pathways has also been acknowledged as part of the complex mechanisms controlling mammalian heart size, with the first antagonizing the nuclear β-catenin/YAP interaction in differentiating cardiomyocytes (Heallen et al., 2011).

Lately, a study by Azzolin and co-workers proposed a new model in which WNT/β-catenin signaling responses are being mediated, in a context-dependent manner, not only by β-catenin activation, but also by TAZ stabilization and TAZ-dependent transcription, thus suggesting TAZ as downstream co-activator of β-catenin target genes (Azzolin et al., 2012). This study also revealed a Hippo inhibitory activity of β-catenin, inducing TAZ inhibition through its interaction with proteasomal complex. Although these findings come mainly from studies on cancer cells, similar interaction might positively or negatively regulate cardiac stem cells where both β-catenin and TAZ have been demonstrated to be instrumental to the self-renewing and differentiation processes.

Another example of how the interaction of known signaling pathways can affect the regulation of cardiac progenitors is given in mouse embryos, where the ablation of Notch1 in Islet1+ CPCs triggers their expansion by negatively regulating Wnt canonical signaling and thus increasing the levels of active β-catenin protein. The subsequent inhibition in their cardiac differentiation is reported (Kwon et al., 2009; Andersen et al., 2012). Further studies will be required to confirm such data in adult cardiac stem cells.

## Conclusions and future perspectives

In conclusion, studying the regulation of adult cardiac stem cell homeostasis and differentiation could require a higher degree of complexity. Few studies have highlighted a complex network of interactions among several signaling cascades contributing to the control of cardiac specification.

**Figure 1:**
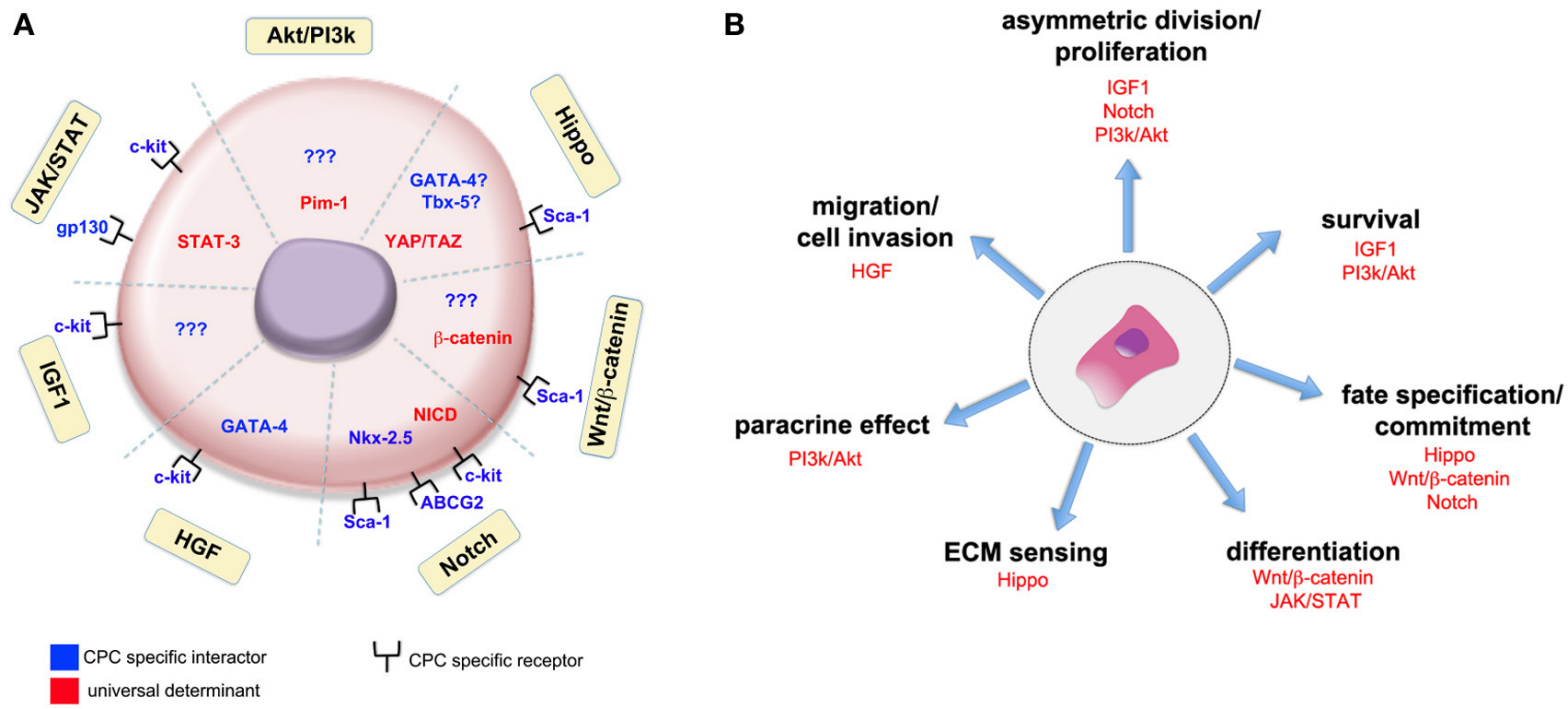
**Universal signaling pathways and cell-specific actuators interact in cardiac stem cells to induce given biological responses**. The interaction of universal determinants (in red) and stage-specific actuators (in blue) in adult cardiac progenitor cells (CPCs) is schematically represented **(A)**. Yellow boxes identify the signaling pathways so far recognized to participate in CPCs function. The association with CPC specific receptors has been found for some of the pathways, while for others no clear evidence of any interaction with cell-specific determinants has been given so far (indicated by question marks). The participation of well-known signaling pathways to given activities in adult cardiac progenitor cells is shown in **(B)**. Please refer to the main text for the meaning of the abbreviations.

Therapeutic approaches trying to enhance the impact of cardiac regeneration or involving the differentiation of resident cardiac progenitor cells will also benefit from the knowledge produced. A graphical representation of such interactions occurring in the context of adult cardiac progenitor cells is shown in Figure 1.

In fact, investigations aimed at highlighting the interaction between universal signaling pathways and stage-specific determinants are overseen as a promising perspective to design novel approaches to the treatment of cardiac diseases. A context-specific system is already considered a dogma as referred to the nucleus of different cell types, where pleiotropic transducers physically interact with cell-specific transcription factors (*adaptors*) to evoke unique responses in terms of gene program activation (Andel et al., 1999).

While apparently multiplying the complexity of the regulatory mechanisms presiding at cardiac stem cell homeostasis, the idea that such a modular interaction exists provides a hub for biochemical and high-throughput studies to unveil the nature and function of such mechanisms and help to design strategies to control cardiac stem cell function in a clinical perspective.

For example, the context-specific activity of intracellular effectors can be selectively tuned by changes in the mechanics of tissue-specific extracellular milieu, like in the case of Hippo pathway transducers affecting cell responses (growth, proliferation and differentiation) through the modification of the cellular genetic program (Mendez and Janmey, 2012; Mosqueira et al., 2014).

Understanding and reproducing this extremely sophisticated level of control will be vital to the success of cardiac regenerative therapies in the next future (Lutolf et al., 2009).

### Conflict of interest statement

The authors declare that the research was conducted in the absence of any commercial or financial relationships that could be construed as a potential conflict of interest.
